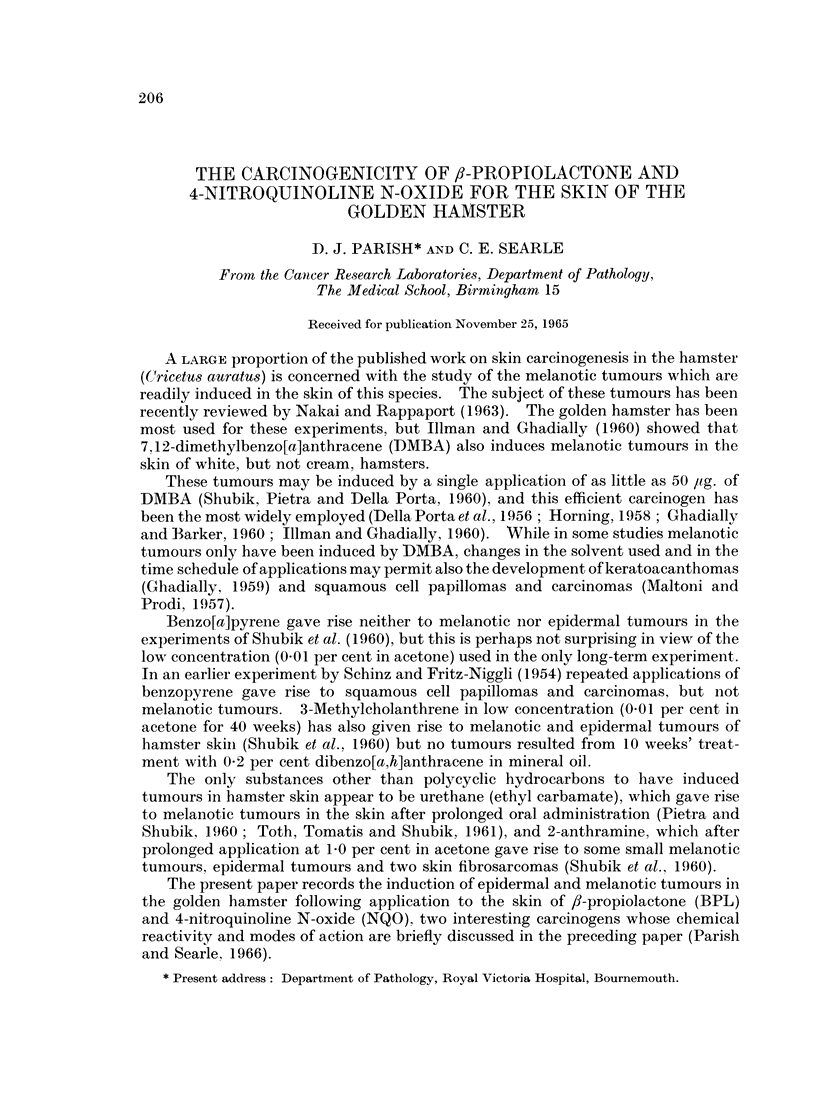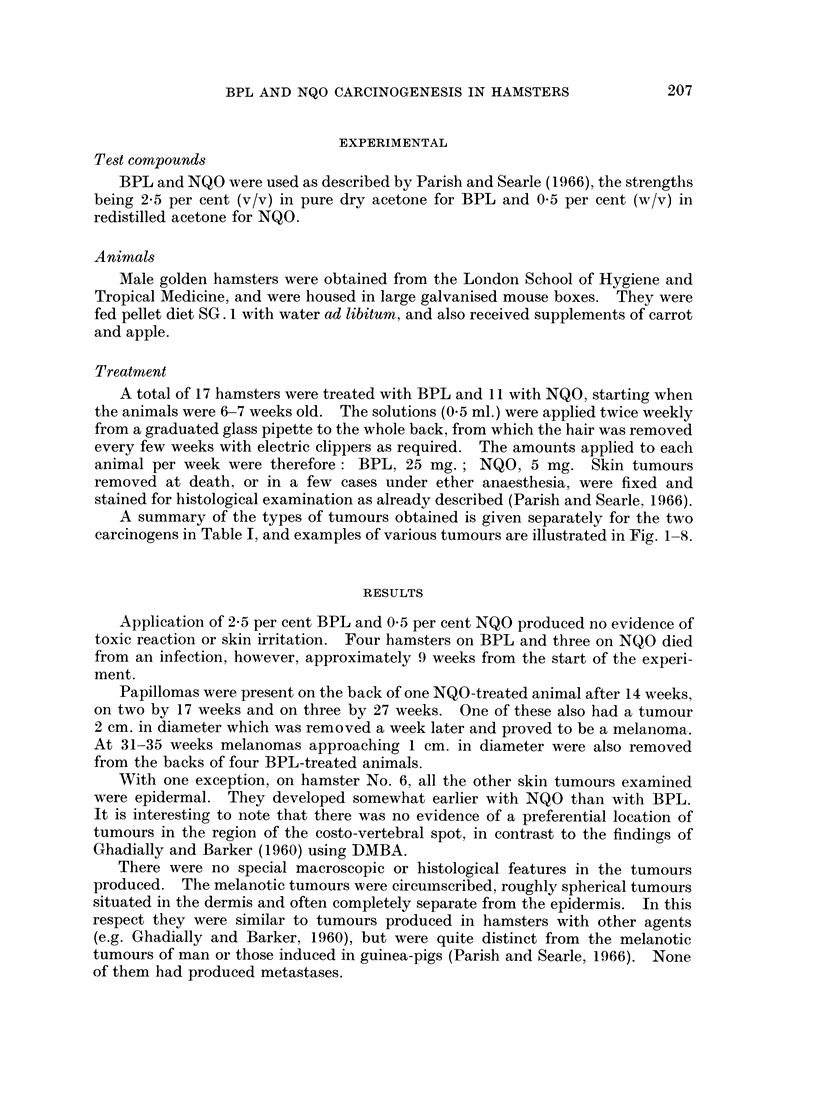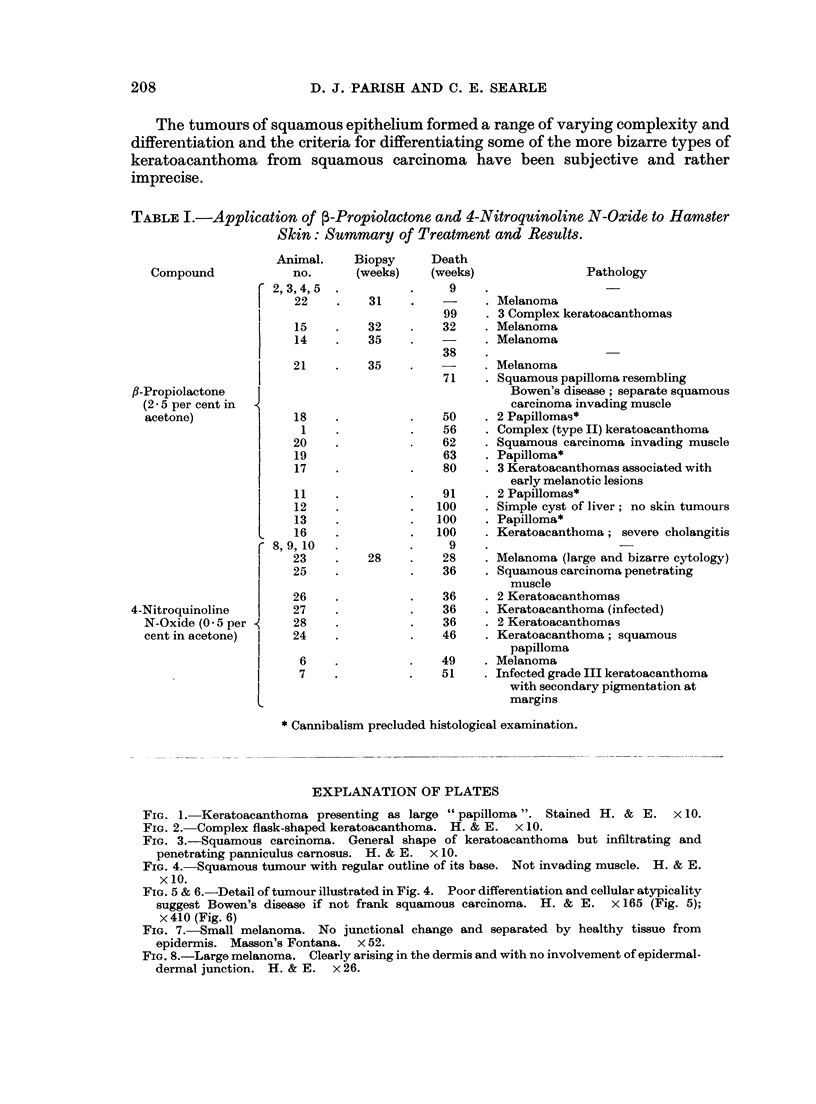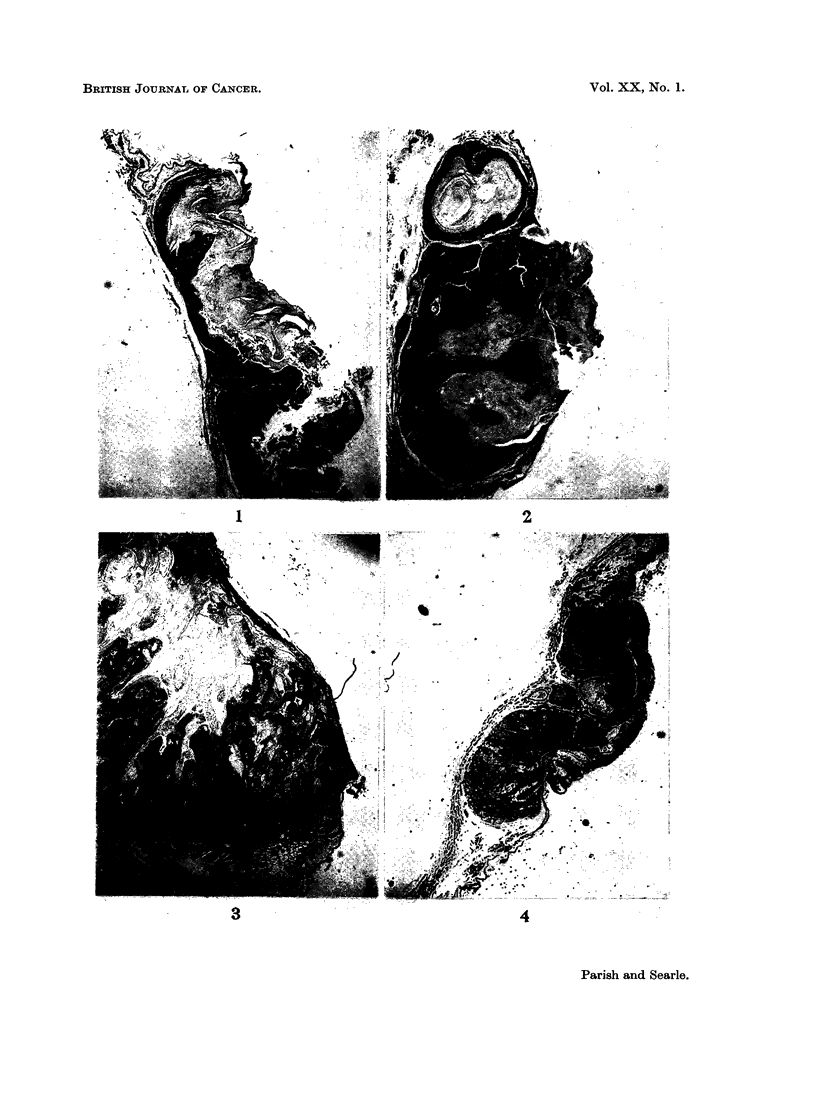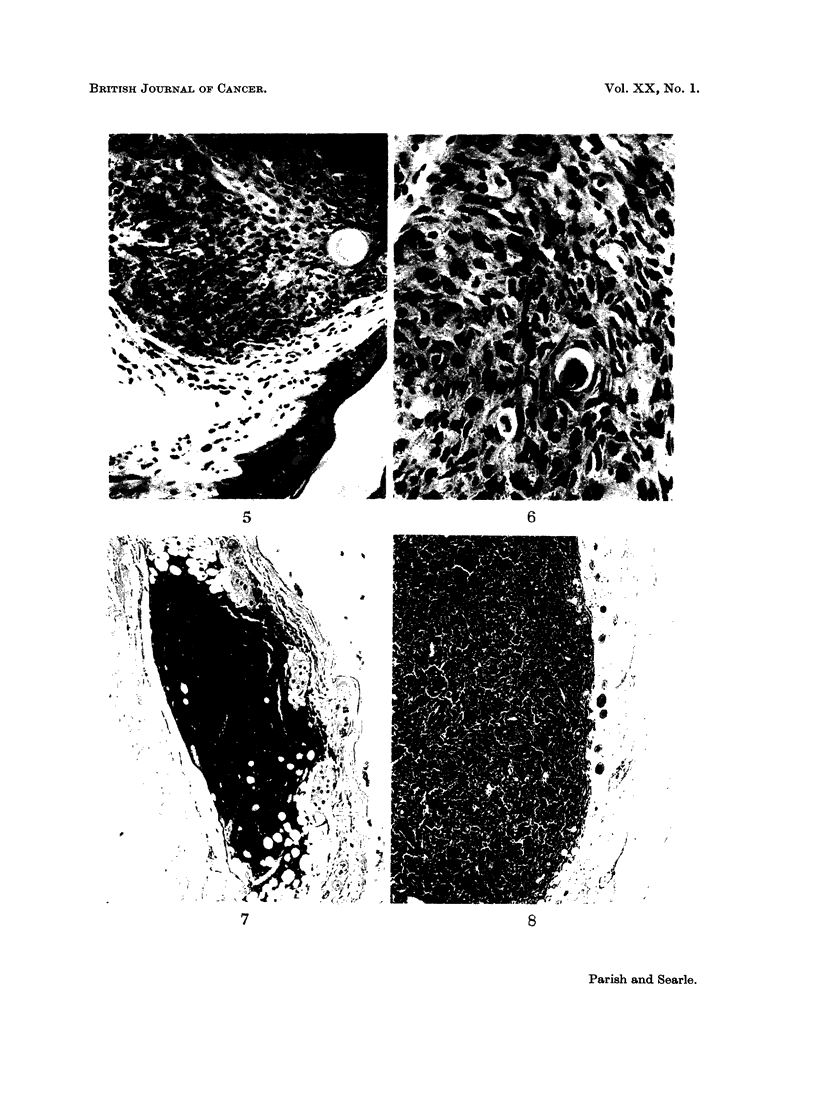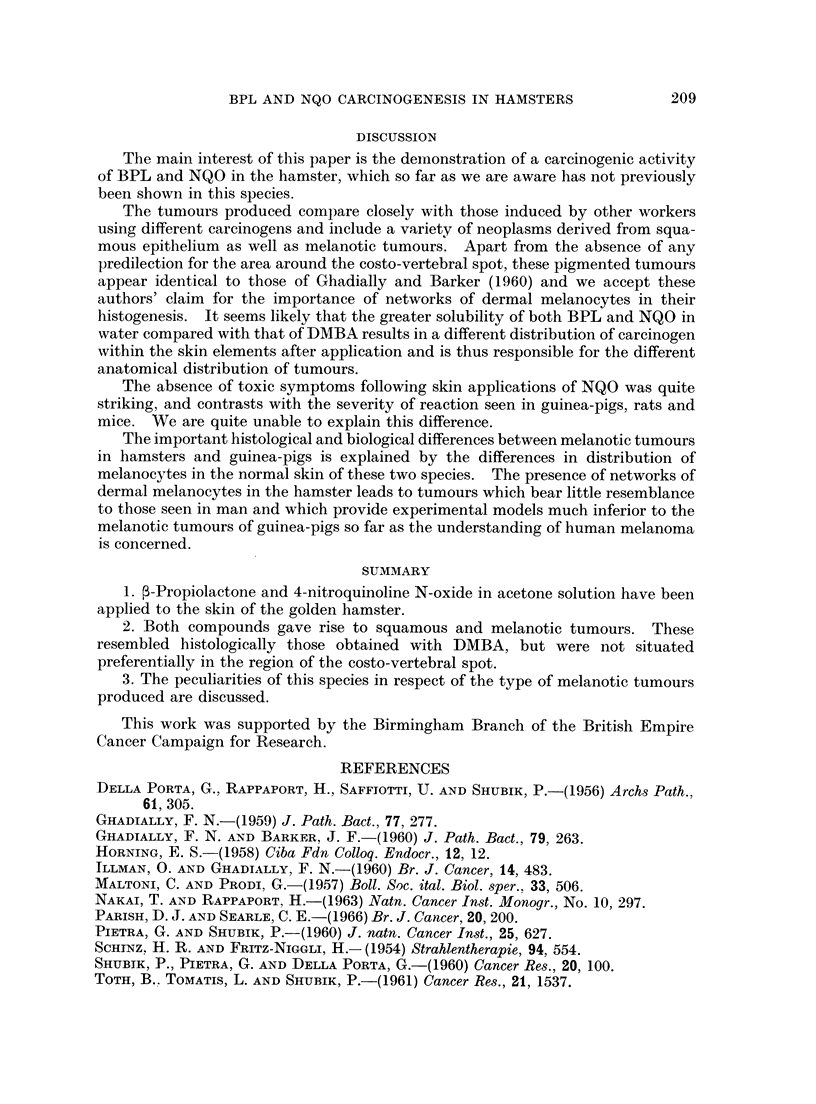# The carcinogenicity of beta-propiolactone and 4-nitroquinoline N-oxide for the skin of the golden hamster.

**DOI:** 10.1038/bjc.1966.24

**Published:** 1966-03

**Authors:** D. J. Parish, C. E. Searle

## Abstract

**Images:**


### THE CARCINOGENICITY OF β-PROPIOLACTONE AND 4-NITROQUINOLINE N-OXIDE FOR THE SKIN OF THE GOLDEN HAMSTER

D. J. PARISH* AND C. E. SEARLE

*From the Cancer Research Laboratories, Department of Pathology, The Medical School, Birmingham* 15

Received for publication November 25, 1965

A LARGE proportion of the published work on skin carcinogenesis in the hamster (*Cricetus auratus*) is concerned with the study of the melanotic tumours which are readily induced in the skin of this species. The subject of these tumours has been recently reviewed by Nakai and Rappaport (1963). The golden hamster has been most used for these experiments, but Illman and Ghadially (1960) showed that 7,12-dimethylbenzo[*a*]anthracene (DMBA) also induces melanotic tumours in the skin of white, but not cream, hamsters.

These tumours may be induced by a single application of as little as 50 μg. of DMBA (Shubik, Pietra and Della Porta, 1960), and this efficient carcinogen has been the most widely employed (Della Porta *et al.*, 1956 ; Horning, 1958 ; Ghadially and Barker, 1960 ; Illman and Ghadially, 1960). While in some studies melanotic tumours only have been induced by DMBA, changes in the solvent used and in the time schedule of applications may permit also the development of keratoacanthomas (Ghadially, 1959) and squamous cell papillomas and carcinomas (Maltoni and Prodi, 1957).

Benzo[*a*]pyrene gave rise neither to melanotic nor epidermal tumours in the experiments of Shubik *et al.* (1960), but this is perhaps not surprising in view of the low concentration (0·01 per cent in acetone) used in the only long-term experiment. In an earlier experiment by Schinz and Fritz-Niggli (1954) repeated applications of benzopyrene gave rise to squamous cell papillomas and carcinomas, but not melanotic tumours. 3-Methylcholanthrene in low concentration (0·01 per cent in acetone for 40 weeks) has also given rise to melanotic and epidermal tumours of hamster skin (Shubik *et al.*, 1960) but no tumours resulted from 10 weeks' treatment with 0·2 per cent dibenzo[*a,h*]anthracene in mineral oil.

The only substances other than polycyclic hydrocarbons to have induced tumours in hamster skin appear to be urethane (ethyl carbamate), which gave rise to melanotic tumours in the skin after prolonged oral administration (Pietra and Shubik, 1960 ; Toth, Tomatis and Shubik, 1961), and 2-anthramine, which after prolonged application at 1·0 per cent in acetone gave rise to some small melanotic tumours, epidermal tumours and two skin fibrosarcomas (Shubik *et al.*, 1960).

The present paper records the induction of epidermal and melanotic tumours in the golden hamster following application to the skin of β-propiolactone (BPL) and 4-nitroquinoline N-oxide (NQO), two interesting carcinogens whose chemical reactivity and modes of action are briefly discussed in the preceding paper (Parish and Searle, 1966).

* Present address : Department of Pathology, Royal Victoria Hospital, Bournemouth.

### EXPERIMENTAL

*Test compounds*

BPL and NQO were used as described by Parish and Searle (1966), the strengths being 2·5 per cent (v/v) in pure dry acetone for BPL and 0·5 per cent (w/v) in redistilled acetone for NQO.

*Animals*

Male golden hamsters were obtained from the London School of Hygiene and Tropical Medicine, and were housed in large galvanised mouse boxes. They were fed pellet diet SG. 1 with water *ad libitum*, and also received supplements of carrot and apple.

*Treatment*

A total of 17 hamsters were treated with BPL and 11 with NQO, starting when the animals were 6–7 weeks old. The solutions (0·5 ml.) were applied twice weekly from a graduated glass pipette to the whole back, from which the hair was removed every few weeks with electric clippers as required. The amounts applied to each animal per week were therefore: BPL, 25 mg.; NQO, 5 mg. Skin tumours removed at death, or in a few cases under ether anaesthesia, were fixed and stained for histological examination as already described (Parish and Searle, 1966).

A summary of the types of tumours obtained is given separately for the two carcinogens in Table I, and examples of various tumours are illustrated in Fig. 1–8.

### RESULTS

Application of 2·5 per cent BPL and 0·5 per cent NQO produced no evidence of toxic reaction or skin irritation. Four hamsters on BPL and three on NQO died from an infection, however, approximately 9 weeks from the start of the experiment.

Papillomas were present on the back of one NQO-treated animal after 14 weeks, on two by 17 weeks and on three by 27 weeks. One of these also had a tumour 2 cm. in diameter which was removed a week later and proved to be a melanoma. At 31–35 weeks melanomas approaching 1 cm. in diameter were also removed from the backs of four BPL-treated animals.

With one exception, on hamster No. 6, all the other skin tumours examined were epidermal. They developed somewhat earlier with NQO than with BPL. It is interesting to note that there was no evidence of a preferential location of tumours in the region of the costo-vertebral spot, in contrast to the findings of Ghadially and Barker (1960) using DMBA.

There were no special macroscopic or histological features in the tumours produced. The melanotic tumours were circumscribed, roughly spherical tumours situated in the dermis and often completely separate from the epidermis. In this respect they were similar to tumours produced in hamsters with other agents (e.g. Ghadially and Barker, 1960), but were quite distinct from the melanotic tumours of man or those induced in guinea-pigs (Parish and Searle, 1966). None of them had produced metastases.

The tumours of squamous epithelium formed a range of varying complexity and differentiation and the criteria for differentiating some of the more bizarre types of keratoacanthoma from squamous carcinoma have been subjective and rather imprecise.

TABLE I.—*Application of β-Propiolactone and 4-Nitroquinoline N-Oxide to Hamster Skin: Summary of Treatment and Results.*

| Compound | Animal. no. | Biopsy (weeks) | Death (weeks) | Pathology |
|---|---|---|---|---|
| β-Propiolactone (2·5 per cent in acetone) | 2, 3, 4, 5 | | 9 | — |
| | 22 | 31 | — | Melanoma |
| | | | 99 | 3 Complex keratoacanthomas |
| | 15 | 32 | 32 | Melanoma |
| | 14 | 35 | — | Melanoma |
| | | | 38 | — |
| | 21 | 35 | — | Melanoma |
| | | | 71 | Squamous papilloma resembling Bowen's disease ; separate squamous carcinoma invading muscle |
| | 18 | | 50 | 2 Papillomas* |
| | 1 | | 56 | Complex (type II) keratoacanthoma |
| | 20 | | 62 | Squamous carcinoma invading muscle |
| | 19 | | 63 | Papilloma* |
| | 17 | | 80 | 3 Keratoacanthomas associated with early melanotic lesions |
| | 11 | | 91 | 2 Papillomas* |
| | 12 | | 100 | Simple cyst of liver ; no skin tumours |
| | 13 | | 100 | Papilloma* |
| | 16 | | 100 | Keratoacanthoma ; severe cholangitis |
| 4-Nitroquinoline N-Oxide (0·5 per cent in acetone) | 8, 9, 10 | | 9 | — |
| | 23 | 28 | 28 | Melanoma (large and bizarre cytology) |
| | 25 | | 36 | Squamous carcinoma penetrating muscle |
| | 26 | | 36 | 2 Keratoacanthomas |
| | 27 | | 36 | Keratoacanthoma (infected) |
| | 28 | | 36 | 2 Keratoacanthomas |
| | 24 | | 46 | Keratoacanthoma ; squamous papilloma |
| | 6 | | 49 | Melanoma |
| | 7 | | 51 | Infected grade III keratoacanthoma with secondary pigmentation at margins |

* Cannibalism precluded histological examination.

### EXPLANATION OF PLATES

FIG. 1.—Keratoacanthoma presenting as large "papilloma". Stained H. & E. ×10.

FIG. 2.—Complex flask-shaped keratoacanthoma. H. & E. ×10.

FIG. 3.—Squamous carcinoma. General shape of keratoacanthoma but infiltrating and penetrating panniculus carnosus. H. & E. ×10.

FIG. 4.—Squamous tumour with regular outline of its base. Not invading muscle. H. & E. ×10.

FIG. 5 & 6.—Detail of tumour illustrated in Fig. 4. Poor differentiation and cellular atypicality suggest Bowen's disease if not frank squamous carcinoma. H. & E. ×165 (Fig. 5); ×410 (Fig. 6)

FIG. 7.—Small melanoma. No junctional change and separated by healthy tissue from epidermis. Masson's Fontana. ×52.

FIG. 8.—Large melanoma. Clearly arising in the dermis and with no involvement of epidermal-dermal junction. H. & E. ×26.

1

2

3

4

Parish and Searle.

5

6

7

8

### DISCUSSION

The main interest of this paper is the demonstration of a carcinogenic activity of BPL and NQO in the hamster, which so far as we are aware has not previously been shown in this species.

The tumours produced compare closely with those induced by other workers using different carcinogens and include a variety of neoplasms derived from squamous epithelium as well as melanotic tumours. Apart from the absence of any predilection for the area around the costo-vertebral spot, these pigmented tumours appear identical to those of Ghadially and Barker (1960) and we accept these authors' claim for the importance of networks of dermal melanocytes in their histogenesis. It seems likely that the greater solubility of both BPL and NQO in water compared with that of DMBA results in a different distribution of carcinogen within the skin elements after application and is thus responsible for the different anatomical distribution of tumours.

The absence of toxic symptoms following skin applications of NQO was quite striking, and contrasts with the severity of reaction seen in guinea-pigs, rats and mice. We are quite unable to explain this difference.

The important histological and biological differences between melanotic tumours in hamsters and guinea-pigs is explained by the differences in distribution of melanocytes in the normal skin of these two species. The presence of networks of dermal melanocytes in the hamster leads to tumours which bear little resemblance to those seen in man and which provide experimental models much inferior to the melanotic tumours of guinea-pigs so far as the understanding of human melanoma is concerned.

### SUMMARY

1. β-Propiolactone and 4-nitroquinoline N-oxide in acetone solution have been applied to the skin of the golden hamster.

2. Both compounds gave rise to squamous and melanotic tumours. These resembled histologically those obtained with DMBA, but were not situated preferentially in the region of the costo-vertebral spot.

3. The peculiarities of this species in respect of the type of melanotic tumours produced are discussed.

This work was supported by the Birmingham Branch of the British Empire Cancer Campaign for Research.

### REFERENCES

Della Porta, G., Rappaport, H., Saffiotti, U. and Shubik, P.—(1956) *Archs Path.*, **61**, 305.
Ghadially, F. N.—(1959) *J. Path. Bact.*, **77**, 277.
Ghadially, F. N. and Barker, J. F.—(1960) *J. Path. Bact.*, **79**, 263.
Horning, E. S.—(1958) *Ciba Fdn Colloq. Endocr.*, **12**, 12.
Illman, O. and Ghadially, F. N.—(1960) *Br. J. Cancer*, **14**, 483.
Maltoni, C. and Prodi, G.—(1957) *Boll. Soc. ital. Biol. sper.*, **33**, 506.
Nakai, T. and Rappaport, H.—(1963) *Natn. Cancer Inst. Monogr.*, No. 10, 297.
Parish, D. J. and Searle, C. E.—(1966) *Br. J. Cancer*, **20**, 200.
Pietra, G. and Shubik, P.—(1960) *J. natn. Cancer Inst.*, **25**, 627.
Schinz, H. R. and Fritz-Niggli, H.—(1954) *Strahlentherapie*, **94**, 554.
Shubik, P., Pietra, G. and Della Porta, G.—(1960) *Cancer Res.*, **20**, 100.
Toth, B., Tomatis, L. and Shubik, P.—(1961) *Cancer Res.*, **21**, 1537.